# Mothers’ Influence

**Published:** 1869-04

**Authors:** 


### MOTHERS' INFLUENCE.

Goethe was a prince of poets and the master-spirit of the age. "From my dear little mother I derive my happy disposition," he records in one of his poems. "Little" as she was, she had an energy and power, a moral force which well befitted the mother of one of the greatest names in Germany.

She says of herself: "Order and quiet are my characteristics. I despatch at once what I have to do—the most disagreeable things first. I always seek out what is good in people, and leave what is bad to Him who made mankind, and who knows how to round off the angles." Decision of character, charity, these two; when you have anything to do, do it. Love; without which all else is as sounding brass, and a tinkling cymbal! Study for these, reader; carry them out legitimately in every department of business and life, and they will make you and yours worthy to be immortal.

It was at the end of his boisterous and belligerent life that the mind of John Randolph, with unutterable emotions of tenderness, wandered back to his mother's knee in the attitude of prayer—"Our Father."

Said Thomas H. Benton: "My mother asked me to promise her that I would never taste a drop of liquor, and I never did;" and few can compute the amount of influence for good which Benton's mind had on our country's destinies. Sometimes it may be that in after life a man may not be able to point his finger to any of the moulding influences of a mother's mind, yet he may remember a few of her sayings, and these never lose their power. "No good ever comes of fooling;" "it 's a long lane that has no turn in it," were all that was remembered of a mother's teachings by one of our public men who has made a mark in his time. Let every mother, then, feel her responsibility, and let her select at least one living, or generous, or noble sentiment, and so engrave it on the memory of her child, that time can never eradicate it, and it will bring forth fruit in the next age, which will bless a family, a neighborhood, a village, a city, a country, or the wide world! Who knows? It was from a mother's counsel that the great Washington "never told a lie." And as the mother of a Samuel, who shall not revere, for all time, the name of HANNAH?

